# Running virtual reality experiments online: A brief introduction and tutorial

**DOI:** 10.3758/s13428-026-03025-w

**Published:** 2026-04-29

**Authors:** Levi Kumle, Alfie Brazier, Joel Kovoor, Johannes Keil, Anna C. Nobre, Dejan Draschkow

**Affiliations:** 1 Department of Experimental Psychology, University of Oxford, Oxford, UK; 2 Division of Psychology and Language Sciences, University College London, London, UK; 3 Faculty of Psychology and Neuroscience, Maastrich University, Maastrich, Netherlands; 4 Wu Tsai Institute and Department of Psychology, Yale University, New Haven, USA; 5 Oxford University Centre for Integrative Neuroimaging, Oxford Centre for Human Brain Activity, University of Oxford, Oxford, UK

**Keywords:** Virtual reality, Online data collection, OnlineVR-toolbox

## Abstract

Achieving meaningful and representative findings in cognitive/behavioural research requires both externally valid experimental settings and representative participant samples. Virtual reality (VR) has become a powerful tool for enhancing realism while maintaining control of experimental environments. Simultaneously, online testing broadens and diversifies samples. Combining these approaches—running VR experiments online—is compelling but remains challenging due to incompatibilities between tools used in lab-based VR studies and online testing. This paper offers a practical solution to bridge this gap. We provide a brief overview of current approaches to online VR experimentation and offer a step-by-step tutorial for converting lab-based VR studies into remote deployments using Unity and Steam. Further, we share an open-source onlineVR-toolbox—including code, example workflows, and practical Notebooks—to facilitate critical processes such as secure data transfer and participant management. To validate this approach, we present a proof-of-concept case study replicating a well-established memory effect. Our results demonstrate that remote data collection via Steam is technically feasible and yields results consistent with laboratory-based findings. Together, our tutorial, tools, and case study show that online VR studies are possible and practical, opening the door to more scalable, inclusive, and externally valid behavioural science.

**Supplementary Information:**

The online version contains supplementary material available at 10.3758/s13428-026-03025-w.

## Introduction

Conducting cognitive studies that accurately reflect real-world behaviours across diverse settings, populations, and timeframes is far from trivial. Traditional laboratory experiments often struggle to achieve external validity due to constrained environments, homogeneous participant samples, and limited capacity for studying behavioural changes over time. These challenges call for innovative methodologies that enhance the representativeness of behavioural research without compromising experimental control.

Virtual reality (VR) has emerged as a powerful tool for conducting more representative studies (Bailenson et al., 2025; Draschkow et al., 2023; Tarr & Warren, 2002). In VR, researchers can fully control the experimental environment while also tracking participants’ field of view, spatial position, and detailed movements of the eyes, hands, body, and head. The ability to precisely monitor participants’ interactions with their surroundings—including their movements, visual attention, and engagement—enables researchers to study fundamental cognitive systems in contexts that closely mirror real-world conditions without sacrificing experimental control. For instance, VR has led to foundational insights into how individuals perceive and navigate their environments (Cohen et al., 2020; Graves et al., 2020; Haskins et al., 2022; Kim & Doeller, 2024; Liu & Ballard, 2021; Maoz et al., 2023; Rothkopf et al., 2016; Siegel & Kelly, 2017; Stokes et al., 2022; Tanrikulu et al., 2023; van Veen et al., 1998; Varshney et al., 2024; Warren et al., 2001; Woolley et al., 2014), search for relevant information in their environment (Beitner et al., 2023; Botch et al., 2023; David et al., 2020; Draschkow & Võ, 2017; Duarte & Geng, 2024; Helbing et al., 2020, 2022; Kristjánsson et al., 2022; Li et al., 2016; Olk et al., 2018), or use their memory to complete tasks (Aivar et al., 2024; Bischof et al., 2023; Chawoush et al., 2023; Diaz et al., 2013; Draschkow et al., 2021, 2022; Helbing et al., 2020; Klinghammer et al., 2016; Klotzsche et al., 2023; Kourtesis et al., 2021; Kumle et al., 2024, 2025; Mynick et al., 2025; Ouellet et al., 2018; Schuetz et al., 2024; Thom et al., 2023). Beyond delivering behavioural insights, VR has the potential to transform clinical treatments (Badger et al., 2023; Deng et al., 2019; Freeman, 2008, 2022; Freeman et al., 2017; Maples-Keller et al., 2017; Rothbaum et al., 2000; van Loenen et al., 2022) and improve learning and education (Di Natale et al., 2020; Fromm et al., 2025; Makransky et al., 2019; Radianti et al., 2020). In summary, VR has been recognised as an effective tool for increasing the external validity of experimental settings.

Beyond the real-world ecological validity of experimental settings, participants also need to be representative of the sample of people to whom the researcher wishes their findings to generalise (Falk et al., 2013; Henrich et al., 2010; Sears, 1986). To reach a larger and more representative participant pool, researchers have adopted remote, online data collection of participants. However, while screen-based (desktop, mobile) online studies have gained traction and become popular, remote online VR studies are still in their infancy.

## Online VR is an efficient solution for conducting representative studies

Unlike traditional online studies, VR allows for precise environmental control while capturing rich behavioural data, such as head and hand movements. For the first time in remote research, researchers will be able to account for participants’ visual field, gaze behaviour, locomotion, and navigation during study completion. This contrasts with traditional screen-based remote studies, where researchers have no control over, for example, visual information or distractions in the participant’s environment. This will allow researchers to construct representative, well-controlled settings, while at the same time profiting from the scalability, fast results, and more representative samples of the online world. As such, online VR can be a powerful tool for studying behaviours in large samples and diverse settings over time, without sacrificing experimental control (Draschkow, 2022).

However, while many dedicated research tools and resources for remote data collection have been developed (e.g., crowdsourcing platforms, experimental software solutions, hosting services), these are largely incompatible with the tools used for in-lab VR studies. As such, despite the promise of remote VR studies (Loetscher et al., 2023; Radiah et al., 2021; Ratcliffe et al., 2021), running them remains challenging in practice.

This resource serves (1) as a brief introduction to online VR experiments and (2) as an accessible, introductory guide on how to migrate an existing *lab-based* VR study into an *online* VR study. Our primary target audience is researchers who already have, or are currently considering, a lab-based VR experiment and would like to use it to collect data online. We assume basic familiarity with implementing VR studies; however, no background or preexisting knowledge about online infrastructure or data-collection setups is required. Hopefully, researchers who are making their first steps into VR-based studies will also find our paper useful as a general introductory resource.

First, we summarise the most feasible approaches for collecting VR data in remote settings and discuss strengths and weaknesses. Next, we present a step-by-step tutorial showing how to collect remote VR data using existing digital distribution services. To aid the development of online VR studies, we also provide an onlineVR-toolbox with accompanying practical Notebooks, code, and resources. In the final section of this paper, we summarise the findings of a proof-of-concept case study that uses Unity and Steam (the most popular VR development platform and distribution service, respectively).

## General approaches for running VR studies online

**Fig. 1:**
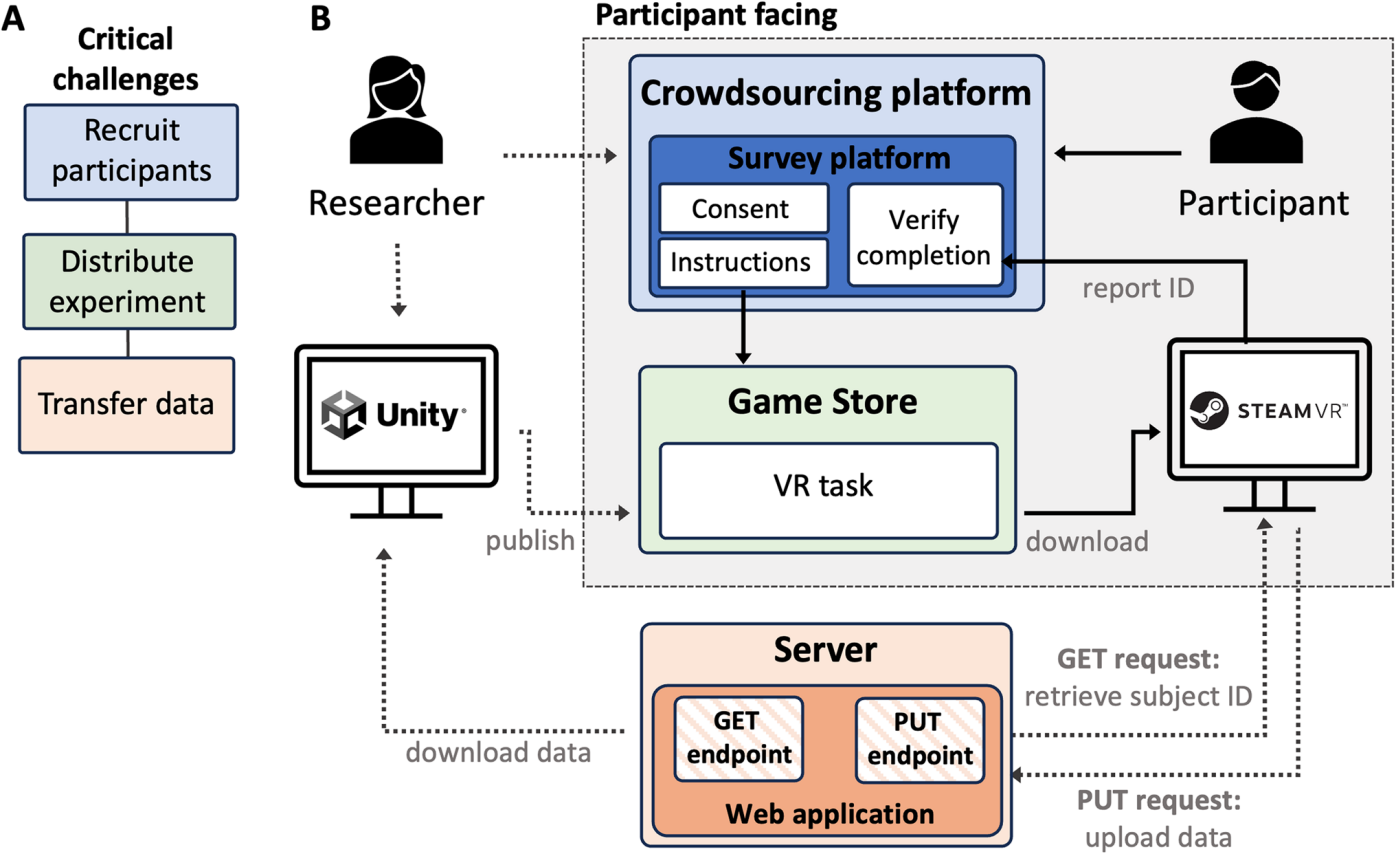
*Overview of challenges and solution for running VR studies online*. **A** In general, conducting online studies involves several steps. Eligible participants must be recruited, and the task must be distributed in a way that participants can access it. Once participants complete the task, any recorded data need to be transferred back to the researchers securely. **B** Pipeline for running VR studies online by distributing them through a digital distribution service (“game store”) commonly used by VR users. Here, researchers can publish their VR application on game stores, which participants recruited through crowdsourcing platforms (e.g., Prolific) can access. Task data can be uploaded though a web application hosted on a server, but this data transfer functionality must be implemented manually

Conducting online studies—whether VR-based or not—requires several key steps (see Fig. 1A) (Sauter et al., 2020). First, participants must be recruited and provided with clear instructions on how to access and complete the study. As a prerequisite for participants completing the study, the experiment must be accessible for remote participation. That is, it needs to be implemented in a format that is compatible with online deployment and distributed on a suitable platform. Finally, as participants engage with the experiment, their data must be securely recorded and transferred back to the researcher.

First, when it comes to participant recruitment, frameworks established for traditional computer-based online experiments can also be exploited for VR-based studies. This includes participant recruitment via crowdsourcing platforms such as Prolific. Specifically, these platforms allow researchers to filter users who own a VR headset. Additionally, case studies suggest that these participants are willing to engage in VR research (Loetscher et al., 2023; Radiah et al., 2021; see also Kelly et al., 2021, for an overview on demographics of VR headset owners). Therefore, existing crowdsourcing platforms provide a viable solution for recruiting participants for online VR studies.

In contrast, when it comes to the implementation, distribution, and data transfer for VR-based online experiments, existing solutions for remote data collection often do not translate effectively to VR research. As a result, most existing approaches to online VR studies bypass or compromise key benefits offered by lab-based VR studies. In this section, we outline existing approaches for solving the key steps of remote VR studies (i.e., implementation, distribution, and data recording and transfer), noting their advantages and drawbacks (also see Loetscher et al., 2023; Radiah et al., 2021). Importantly, effective solutions should provide researchers with full experimental control and flexibility in implementation, including the ability to implement fully immersive VR applications, and full access to behavioural data recordings.

One approach in remote VR research is the use of social VR platforms such as *VRChat* or *Rec Room* (e.g., Saffo et al., 2020, 2021). These platforms provide virtual spaces where users can interact in real time using avatars. Researchers can upload their VR task to these platforms, which allows them to easily distribute the experiment to their user base. These platforms, however, are not research-focused and are also not compatible with VR development tools widely used in lab-based VR research (e.g., Unity, Unreal, and Vizard). As a result, researchers are constrained by the functionalities offered by these platforms, quickly leading to significant limitations regarding data recording and transfer capabilities, flexibility in experiment design, and experimental control. Consequently, while social VR platforms can be useful in some contexts, they seem an impractical and non-universal solution for adapting most lab-based VR studies for online testing.

Given the promise of remote online VR research, dedicated tools and frameworks are emerging to address its unique challenges. The most notable example is *Ouvrai*, an integrated browser-based solution that provides end-to-end support for VR studies (Cesanek et al., 2024). That is, Ouvrai offers a framework for implementation, data management, and hosting/distributing that can easily be combined with crowdsourcing platforms like Prolific. This is a significant step forward, and we anticipate further developments in VR-specific online research tools. However, switching to new software solutions can be daunting, especially if lab-based implementations already exist. That is, while recruitment and distribution of online VR studies is streamlined by end-to-end solutions, implementing the study can come with a start-up cost and might be constrained by the available capabilities of a specific framework.

An alternative to relying on dedicated end-to-end solutions is the distribution of stand-alone VR applications that have been implemented using the same tools used to power lab-based VR studies (e.g., Unity, Vizard, Unreal Engines, and associated toolboxes; see, e.g., Alsbury-Nealy et al., 2022; Kasowski & Beyeler, 2023; Schuetz et al., 2023; Vasser et al., 2017). Capitalising on such established infrastructure allows researchers to build on existing skills, already implemented lab-based tasks, and the documentation and support available for these widely used tools.

A simple way of distributing a stand-alone VR application is to send it to participants directly (e.g., through a download-link or email), sidestepping the need to host the application (i.e., making it available online). This can be accomplished by generating an executable file that participants download, install, and run on their own devices (see Zhao et al., 2021). However, this requires participants to install unfamiliar, custom software, which can be error-prone, time-consuming, and may discourage participation (it should set off alarm bells for anyone who has ever sat through an information security training). Additionally, transferring experimental data from the participant back to the researcher is not straightforward and needs to be implemented manually.

An alternative established approach for hosting and distributing VR applications is through digital distribution services (“game stores”) commonly used by VR users (see Evans et al., 2023, for a case study) (see Fig. 1B). The popularity of these game stores means that there are extensive online tutorials and protocols available to guide researchers in converting their VR projects (e.g., implemented in Unity or Unreal) into a publishable format. Furthermore, since most commercial VR games and applications are distributed via these platforms, participants are already accustomed to using them. Participants thus run the experiment in a familiar, preconfigured environment, which can help minimise technical issues. Yet, this approach also presents distinct challenges. Most critically, these platforms are not optimised or developed for research. While data transfer functionality and integration with crowdsourcing platforms is possible, it is not built-in and must be implemented manually by the researcher. Additionally, using these platforms entails additional administrative effort, such as getting experiments approved and published. Nevertheless, this approach provides a viable alternative to dedicated end-to-end solutions, especially in scenarios where researchers want to rely on the capabilities and advantages of established VR engines such as Unity.

## Running an online VR study using a digital distribution service: Step-by-step tutorial

As summarised above, distributing VR experiments via an available digital distribution service (e.g., Steam Store) brings key advantages. In this tutorial, we demonstrate that despite some challenges, it is feasible to run online VR studies in this way. Specifically, we provide a step-by-step tutorial showing how to run a VR study programmed in Unity through the Steam store. We have selected these services because Unity is among the most popular software platforms for creating VR experiments, and Steam is the most popular digital distribution service for VR games. Additionally, Steam provides wide hardware support, which facilitates recruitment. However, we expect that most of the steps described will generalise to other software development tools and distribution services. For example, Vizard or Unreal Engine provide helpful companion pieces for publishing projects to the Steam and Oculus stores.

We first provide a conceptual overview of the steps involved to give researchers an idea of the overall process. Additionally, we provide an onlineVR-toolbox consisting of open materials, code, and practical Notebooks which target anticipated challenges. We hope this enables researchers to replicate the proposed pipeline (see Fig. 1B), enabling them to move their VR experiments online.

### Step 1: Implement the VR task in a publishable format

When collecting data in the lab, researchers often run VR tasks directly within the Unity editor. This approach is convenient, as it is easy to configure details (e.g., subject numbers), monitor potential error messages, and track participants’ progress throughout the task. However, to distribute the VR task remotely, we need to package it into a *build*. This process compiles the assets and code from the project into an executable file, creating a stand-alone version of the experiment that can be shared.

Compiling a Unity project into a build is simple and can be done directly within the Unity Editor. However, for researchers accustomed to running tasks within the Editor, there are a few important adjustments to consider for the build to run smoothly, particularly regarding data paths and file access. We summarise common considerations and pointers for creating a build in Notebook 4.

### Step 2: Set up data transfer functionality

Most importantly, we need a way to transfer any recorded task data back to us researchers. This functionality is typically integrated and automatised by research-focused online testing services (e.g., Pavlovia), where researchers simply download the data from the platform of choice without needing to think about how the data arrived there.

Steam, however, is not a research-focused platform and therefore does not provide such services. Therefore, we need to implement data transfer functionality ourselves. We can achieve this by enabling our Unity task to communicate with a remote web application (i.e., a program that runs on a server and can be accessed through a web browser or another client, such as Unity) which handles the transfer and storage of data (see Fig. 1B). This necessitates creating and hosting such a web application. Additionally, the Unity implementation of our VR task must be able to connect to this web application and upload the experiment data.

Developing this necessary infrastructure is not an expected core skill of behavioural researchers and can therefore constitute a major challenge. To make this step much less daunting, in this tutorial we provide a preconfigured pipeline that can easily be integrated into existing Unity projects. In addition to summarising the necessary high-level conceptual steps, we share code and step-by-step Notebooks on how to implement this functionality (see onlineVR-toolbox and Notebooks 1–3). The aim of these practical resources is to provide a toolbox that enables researchers to easily set up the required data transfer functionality and to further build on our solutions.

**Creating a web application:** As mentioned above, we first need to create a web application with the required functionalities (i.e., receiving and storing data). As we will later explore, our VR task might also need to *fetch* data—for example, unique subject ID or encryption key—from our web application. This requires the web application to keep track and update data (i.e., counter of assigned subject numbers), which then can be retrieved by our VR task. Fortunately, these are core capabilities of web API (application programming interfaces), and we can build on existing frameworks to provide us with the required tools. Multiple such web frameworks exist (e.g., Bottle, Django, Flask), each with different strengths and functionalities.

As part of this tutorial, we chose Bottle (Hellkamp, 2022), a lightweight Python web framework that allows the creation of web applications with minimal code and within a single Python script. As part of the onlineVR-toolbox, we provide a preconfigured Bottle web application (also see Notebook 2 for details) which can both allocate unique subject IDs and store received data. In technical terms, we created a “GET endpoint” and “PUT endpoint”, which act as points of contact between the client (i.e., the VR task on a participant’s computer) and our application (see Fig. 1B). The VR task can send requests to these endpoints to retrieve (GET) or transfer (PUT) data.

**Hosting the web application:** If we want our VR task to be able to communicate with our web application (i.e., request the upload or retrieval of data), we need to host the application (i.e., the Python script implementing the application) somewhere where it is accessible over the internet. That is, we need a server that hosts our web application (also see Fig. 1B).

In principle, a server could be any computer. For maximal control and oversight, a computer within the university network could be used to host the server. Configuring and maintaining the server ourselves, however, takes additional technical expertise and may incur liabilities, for example, for data protection. Further, the available hardware can affect the potential performance and availability of the server (e.g., limited bandwidth, dependence on local network reliability). To avoid such challenges, we can make use of third-party services that provide the necessary infrastructure. By providing a cloud-based and preconfigured environment, such services streamline the hosting of web applications.

As part of this tutorial paper, we provide step-by-step instructions (see Notebook 2) on how to host the preconfigured Bottle web application on *PythonAnywhere (*https://www.pythonanywhere.com/*)*, which allows hosting a simple web application for free. PythonAnywhere provides a web-based interface for configuration, hosting, and monitoring of our web application, as well as disk space for temporarily storing the received data.

**Enable VR task to communicate with web application:** So far, we have covered the implementation of the data transfer *backend* (i.e., web application on server). However, our VR task (i.e., client or data transfer *frontend*) also needs to be able to send the appropriate requests to interact with the specified points of contact on the backends’ side (see Fig. 1B). As mentioned earlier, this consists of sending a “GET request” to the “GET endpoint” to retrieve, for instance, a unique subject ID at the start of the experiment. Once the participant has finished the experiment, the VR task then needs to send a “PUT request” containing the task data to the “PUT endpoint”.

Communication with web servers is a common feature required by game developers (e.g., uploading/downloading leaderboard scores or game settings). As such, Unity provides dedicated methods to implement this (see *UnityWebRequest* class). As part of the onlineVR-toolbox, we provide the required functionalities within the onlineVR Unity package. Additionally, we provide step-by-step instructions on how to easily integrate the provided functions within existing Unity projects and the overall experiment logic (see **Notebook 1**).

As a general suggestion, we recommend uploading task data in small batches throughout the experiment (e.g., after every block or trial) to avoid long upload times due to large file sizes and to reduce the risk of data loss in the case of, for example, temporary loss of internet connection or technical errors that prevent participants from completing the study protocol. Further, file sizes and upload speed should be evaluated during piloting to determine a suitable upload strategy. To prevent data loss due to participants closing the experiment before the data have been uploaded successfully (also see “Case study”), we recommend implementing a waiting screen instructing participants to keep the task running until the data have been uploaded successfully.

**Ensuring data protection:** Lastly, ensuring compliance with data protection regulations is essential when implementing data transfer functionalities. As a general principle, encryption should be applied during both transmission and storage on the server to safeguard participant data. Data should only be decrypted by authorised researchers offline, that is, after it has been downloaded from the server.

Most modern programming languages, including C# and Python, provide built-in encryption libraries, making it straightforward to implement secure data handling. As part of the onlineVR-toolbox, we provide an implementation for both encryption and decryption using AES (Advanced Encryption Standard) (see **Notebook 3**).

Finally, while PythonAnywhere provides infrastructure that can be configured in a General Data Protection Regulation (GDPR)-compliant manner, depending on the sensitivity of the task data and data protection and privacy policies, it may not be permissible to use third-party web hosting services. Whether third-party web hosting services (or online VR data collection in general) are a viable option could depend on both the type of data collected by the VR task (e.g., anonymous vs sensitive or personal data) and regional differences in data protection regulations. In general, the proposed pipeline is optimised for studies collecting anonymous data. Within our proposed approach and provided resources, all transferred data are fully anonymous (i.e., no link to Prolific IDs) until they are manually linked on a researcher’s device. However, studies involving identifiable or sensitive clinical information within the VR data itself may require additional safeguards or alternative hosting solutions. In any case, we strongly recommend consulting the relevant ethics committees and data protection officers to ensure compliance with regulatory and institutional requirements when choosing a hosting solution.

### Step 3: Publish experiment on Steam Store

Once we have an online-compatible VR task, we need to make it available on the Steam store (or another chosen game store). This involves several steps and requirements. Given the popularity of Steam, these are clearly laid out in resources provided by Steam. In light of the extensive documentation readily available, here we focus on summarising the most relevant points for researchers. We strongly advise readers to familiarise themselves with the most up-to-date version of the documentation.

**Developer account with Steamworks:** First, a developer account with Steamworks is required to publish on the Steam Store. A developer fee (currently £100) is applied for each “product” you wish to distribute. While it is possible to distribute different versions of the same task (e.g., for counterbalancing—see Step 4 below—or follow-up experiments) within the same product by uploading updated/different versions of the VR task, this cost needs to be factored in for each distinct project that cannot be reasonably distributed in the same “store page” (see below). Importantly, when first creating an account, Steamworks currently imposes a 30-day waiting period between paying the developer fee and distributing the VR task. To avoid delays, it is recommended to set this up early in the development cycle.

**Setting up a “store page”:** Since the experiment will be publicly available for any user on Steam while active, it needs a public-facing “store page” that blends in with other available commercial products. This currently includes a logo, trailer, description, and “game” screenshots. While these do not need to match the quality of commercial games available on these platforms, Steam requires developers to adhere to their guidelines. Store pages need to be submitted for review before the VR task is made available. The review might take several days and might return specific requests for corrections. As part of the case study reported below, the reviews we received were minor and actionable but additionally delayed the release of our experiment. Importantly, the store page can be prepared during the 30-day waiting period (for the developer account) to avoid further delays.

**Preparing the application for release:** After uploading the VR task to Steam (see step-by-step instructions and video tutorials provided by Steam), the application needs to be prepared and approved for release. This includes a “coming soon” sign which needs to be available for 2 weeks. Also, the application will be tested by Steam to see whether everything is running as it should.

### Step 4: Recruiting via crowdsourcing platforms

As noted above, established crowdsourcing platforms like Prolific can be effective for recruiting participants, even for online VR studies (also see “Case study”). When recruiting for VR studies, it is important to filter for participants who have access to the required hardware. Prolific currently allows one to filter participants who own a VR headset. However, when distributing an experiment through a game store like Steam, several additional factors must be considered. First, it should be clearly stated that participants need a *Steam-compatible* headset, must have an active Steam account, and must be able to set up their hardware and access the Steam store to complete the study. Additionally, unlike typical online studies where participants can be directed straight to the task, our approach requires them to independently locate and launch the study on Steam. Thus, additional steps are required to provide step-by-step instructions on how to locate and access the experiment, and to verify completion.

**Linking to Steam and obtaining consent:** One possibility for providing instructions on how to locate and access the VR task on Steam is to use an intermediate online survey platform (e.g., Pavlovia, Qualtrics) to which participants can be directed (for example instructions, see shared materials for the case study). This solution additionally allows for informed consent to be obtained and securely stored before participants proceed with the VR task. Additionally, it provides an opportunity to collect demographic information and other relevant data prior to the experiment, or to provide detailed instructions on the task and study procedure. Alternatively, it is possible to embed the informed consent procedure and instructions for the experiment within the VR task itself (see also “Managing public access” below).

**Implementing counterbalancing:** Given the lack of integration of research-specific recruitment platforms and Steam, special consideration needs to be given to solutions for counterbalancing in the absence of dedicated tools. If an equal number of participants need to experience the same conditions or order of conditions, and randomisation is not sufficient, the current most straightforward approach is to create multiple versions of the same VR task, each with a hard-coded condition order. As discussed in Step 3, setting up different versions of the same task on Steam is straightforward, enabling data collection in batches across different conditions by changing the version that is currently active. Otherwise, counterbalancing can be based on information retrieved from the web application (e.g., subject number), but this may be less reliable, for example, in case participants start the task multiple times while setting up their equipment (each time being assigned a new subject number).

**Verifying completion:** Just as participants must navigate to the experiment on Steam independently, they also cannot be automatically redirected back to the recruiting platform (e.g., Prolific) to confirm completion. Therefore, an alternative method for verifying completion is needed to ensure proper compensation of participants. A simple and effective solution is to display a memorable completion code at the end of the experiment, which participants can then report. For instance, participants can be instructed to return to the intermediate survey platform upon completing the VR task to enter the completion code.

**Linking data across platforms:** Lastly, accounting for how data will be linked across platforms (e.g., Prolific IDs, consent provided on online survey platform, data from VR task) is crucial. While many established online survey platforms provide integrations with Prolific (i.e., IDs are carried forward automatically), matching data from survey or task platforms with the VR data is less automated. However, it is important for multiple reasons. First, compliance with data protection regulations generally means that we need to be able to withdraw data if participants request this. This is only possible if we can link data to specific participants. Second, researchers might collect additional information (e.g., demographics, responses to questionnaires, post-task surveys) that they want to link to participants’ performance in the VR task.

Linking data across platforms can be done in multiple ways. For instance, participants can be provided with unique access keys (e.g., structured self-generated ID code) that participants are prompted to enter at each stage (i.e., survey platform and VR task). Otherwise, participants can be provided with unique completion codes (e.g., based on the subject number queried from the web server, see “Case study”), which they enter within the Prolific integrated survey platform, and which are also stored in their task data and can therefore be used as a link. This is a potential weak point in our pipeline, as participants could make errors in reporting these codes. In this case, any data that cannot be clearly linked would need to be discarded, potentially resulting in some data loss.

**Managing public access:** Since the experiment is distributed on a public platform, anyone could potentially access and complete it. On one hand, this can be a valuable tool for public engagement. On the other hand, a method is needed to distinguish formally recruited research participants from general users, especially if informed consent is obtained before participants navigate to Steam.

Several options are available to differentiate between user types, building upon methods used to link data across platforms. If unique completion codes are used, any data that do not match a reported code can be discarded. If the code is embedded in the filename, filtering can be done before downloading and decrypting the data. Alternatively, if an access-key system is implemented, data may only be uploaded if the entered key matches predefined criteria, removing the need to discard data from general users.

In any case, we recommend explicitly stating on the store page that the VR task is part of a research study, and that while anyone is welcome to explore the VR experiment, only those who have been formally recruited or contacted by the research team will be included in the study. This ensures that general users are aware that their data will not be stored or used for research purposes.

## Case study

**Fig. 2:**
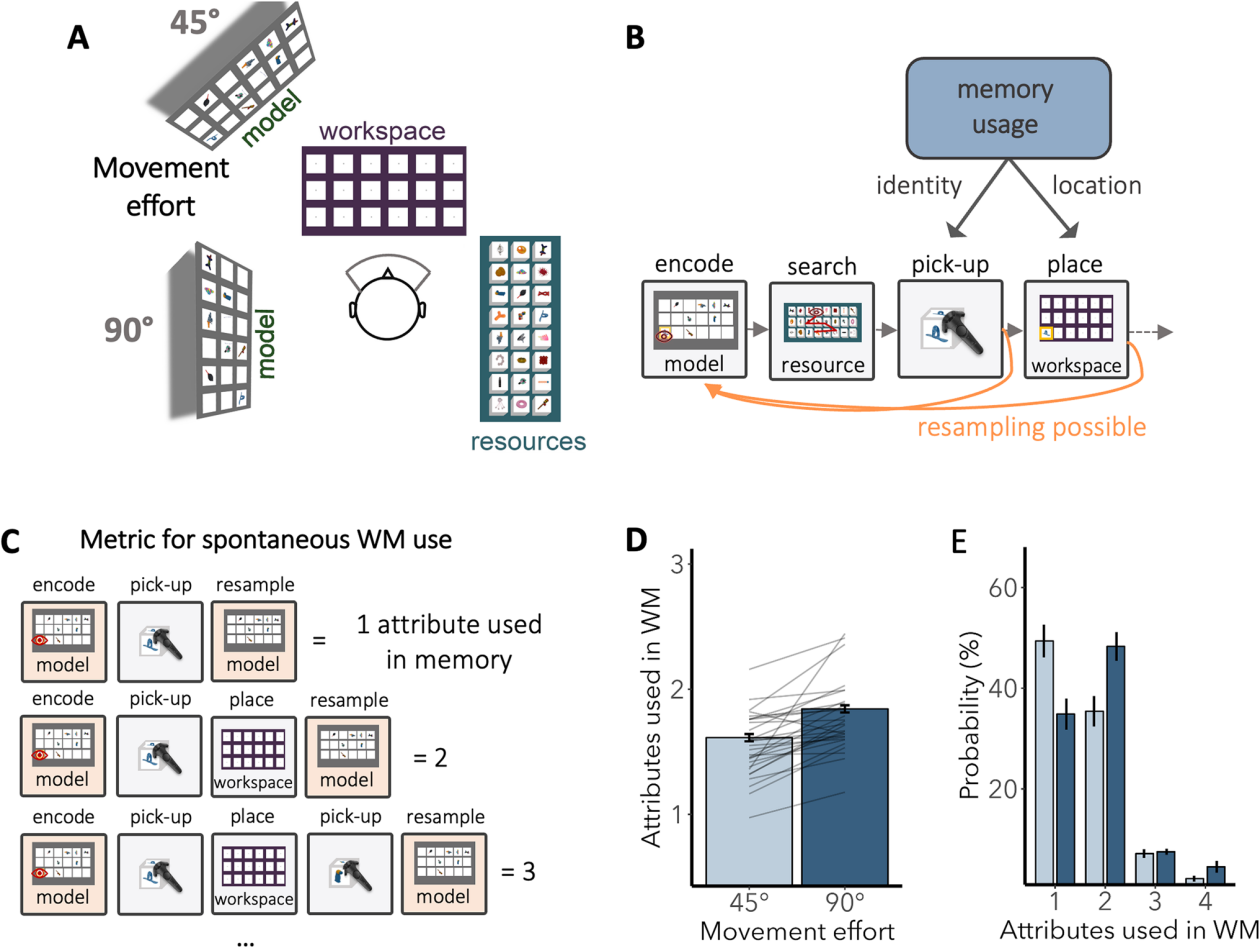
*Methods and results of case study using the* o*bject*-c*opying* t*ask.* **A** Participants were instructed to copy an arrangement shown in the “model display” by picking up objects from the "resource table" and placing them in the corresponding locations on the "workspace". The task included two movement effort conditions (see Video 1). Specifically, the model was positioned either 45° (low movement effort) or 90° (high movement effort) away from the workspace. **B** Task-relevant sensory information remained accessible in the external environment, allowing participants to freely structure their behaviour. Specifically, participants could refer back to the model display at any point during the task (highlighted as “resampling possible” with orange arrows), whether after selecting an object from the resource pool or after placing it in the workspace. **C** An implicit metric for memory usage. Successfully copying an object requires using two attributes: identity for pick-up and location for placement. Counting successful pick-ups (i.e., identity attribute used) and placements (i.e., location attribute used) between model viewings provided a metric for the number of attributes used in memory.** D** Replicating previous research, participants in the online sample used memory more when movement effort was high. **E** When movement effort was low (45°), participants most often used only one attribute on memory. In contrast, when movement effort increased (90°), participants relied on two attributes in memory in most cases (also see Table S1)

To assess the feasibility of the proposed approach, we conducted a case study using the resources detailed and shared above. To highlight the key advantages of the proposed pipeline, we purposefully selected a complex and data-intensive task, which would have been challenging to implement using existing online VR solutions. Specifically, we used a VR adaptation of the object-copying task, a well-established task for studying spontaneous memory use (see Fig. 2A and B) (Ballard et al., 1995; Draschkow et al., 2021; Kumle et al., 2024, 2025).

In the object-copying task, participants are instructed to replicate an arrangement of objects displayed in a “model” by picking up objects from a “resource pool” and placing them in the corresponding locations within a “workspace”. Successful task completion requires encoding task-relevant sensory information (i.e., target identities and locations) from the model display and subsequently using this memory content to guide object pick-up and placement (Fig. 2B and Video 1). Crucially, participants can freely structure their behaviour. For example, they can decide when to look back at the model and in what order to copy the objects. A well-documented effect in in the object-copying task is that increasing the effort required to encode from the model encourages greater reliance on memory (Draschkow et al., 2021; Hardiess et al., 2011; Kumle et al., 2024; Liang et al., 2025; Somai et al., 2020). A useful analogy is cooking with a recipe: If the recipe book is open next to you, you might check it after every step. However, if someone moves it to another room, you are more likely to memorise and then execute multiple steps at once to avoid the inconvenience of repeatedly checking the recipe book.

In this case study, we aimed to replicate this well-established effect, demonstrating both the feasibility of online VR data collection in general and the suitability of our proposed pipeline in particular. Below, we report the findings of our case study alongside general impressions and an evaluation of the proposed approach.

**Recruitment and sample.** Participants were recruited through Prolific, and *N* = 40 participants completed the study protocol (*M*_age_ = 32.25, range_age_ = 21–59; 36 male, 4 female, 0 other/prefer not to say). Participants provided informed consent and received £8 as compensation. The study protocol was approved by the Central University Research Ethics Committee, University of Oxford (#R81997/RE001).

Overall, while recruitment on Prolific was slightly slower than in a comparable screen-based study, we did not encounter significant difficulties in recruiting participants. We collected data in small batches of 5–10 open slots at a time to closely monitor submissions and feedback. Even so, we successfully recruited 40 participants in under 3 days. Notably, more than 400 participants initially accepted the study on Prolific but later returned or timed out their submissions without having attempted to complete the study protocol. This was the primary factor slowing recruitment, as these incomplete submissions temporarily occupied the limited available slots. Importantly, while Prolific allows researchers to filter participants based on them indicating that they *own* a VR setup, this does not guarantee that participants *have access* to their VR setup when the study is advertised to them. That is, many participants may have accepted the study and only afterwards realised that they were currently unable or unwilling to assemble their VR setup. Further, four participants attempted to run the task but encountered unexpected technical difficulties (e.g., controller not recognised by task) which could not be resolved even though the participants reported having a working Steam-compatible VR setup. These participants received partial payment.

For analysis of participants’ feedback on the overall study procedure, responses of all 40 participants who successfully completed the protocol were considered. For analysis of the VR task data, four participants had to be excluded due to an error during data transfer: We only received partial data from these participants, likely because the task was closed by the participant before the upload of data from the last block was complete. As a result, we recommend implementing a waiting screen, instructing participants to keep the task running until the data upload is confirmed.

Six additional participants were excluded based on their task performance. They completed fewer than seven trials before trial timeout in at least one condition, resulting in a final sample of *N* = 30 for task analysis. The sample size for the task analysis was guided by a power analysis using the pwr package (Champely, 2020), informed by Draschkow et al. (2021), as the case study constitutes a direct replication. Based on the effect size for the comparison of 45° versus 90° movement effort reported in Draschkow et al. (2021), reduced by 20% to ensure conservative sample size planning, a sample of 30 participants yielded over 95% power for a paired-sample *t*-test.

**Procedure.** Participants recruited from Prolific were directed to Qualtrics, where they provided informed consent and demographic information (age, gender, handedness). Participants additionally had to confirm that they had a Steam account and a Steam-compatible VR headset, and were prepared and able to set up their VR equipment to complete the study.

Then, participants read through instructions on how to start the experiment through the Steam store (see *Instructions* in shared materials). Specifically, participants were provided with a direct link to the experiment’s Steam Store page and the required search terms, as well as an image confirming what the store page should look like. They were then instructed to set up their VR equipment as they usually would. We additionally provided instructions on the general logic of the task, and what to expect when clicking the Play button. Participants were instructed to keep their Qualtrics survey open while completing the VR experiment.

Participants then started the VR task. Once the experiment successfully established a connection to the remote server, participants first completed a short interactive practice section which repeated the instructions for the VR task and allowed participants to familiarise themselves with the object-copying task (Video 2). Participants then completed two blocks of 12 trials each, where the first two trials in each block served as additional practice trials. Between blocks, we manipulated the movement effort required to encode from the model (see Fig. 2A and Video 1). That is, the model was positioned either 45° (low movement effort condition) or 90° (high movement effort condition). Participants did one block in each movement effort condition, and the order of blocks was counterbalanced between participants. At the end of the VR task, a unique six-digit completion code was displayed to the participant (e.g., 000098), and they were instructed to return to Qualtrics and enter the code. The unique completion code equalled the subject number that was retrieved from a remote server at the beginning of the task (see Notebook 1 and 2) and was stored within the VR task data. Besides verifying completion, it therefore also allowed us to match prolific IDs to the VR data.

Finally, participants filled in a short debriefing questionnaire, where we asked about their feedback on the study procedure (e.g., navigating to Steam) and details about their VR setup (e.g., type of headset).

**Apparatus and virtual environment.** The virtual environment and task were programmed in Unity (version 2022.3; Unity Technologies) using the Steam VR plugin (see *Supplementary Notes 1* for a detailed description of the virtual environment). The task was distributed through the Steam Store under the name “Sort It!”. Data transfer functionalities were implemented using a custom web application hosted on pythonanywhere.com and the *UnityWebRequest* class. Participants used their own commercial VR headsets (Meta Quest, *N* = 32; Oculus Rift, *N* = 3; HP Reverb, *N* = 2; Pimax Crystal, *N* = 1) and one hand-held controller in their dominant hand.

Data were recorded on a frame-by-frame basis. Participants’ continuous head and hand movement was tracked and recorded using the position and rotation data from the VR headset and hand-held controller. Additionally, all interactions with the virtual environment (i.e., grabbing, holding, and placing of objects) were logged. In the absence of eye-tracking capabilities in most consumer-grade VR headsets, we approximated and recorded participants’ gaze on key task areas (the model, workspace, and resource table) by intersecting the head orientation vector (i.e., a unit vector extending forward from the headset’s position in the direction the headset is facing) with these regions of interest. Approximated gaze data were also logged continuously on a frame-by-frame basis.

Importantly, the pipeline supports highly customisable data acquisition comparable to single-participant lab-based setups, including continuous and high-volume recordings. In practice, data recording in the case study was primarily limited by participant’s hardware (e.g., absence of eye-trackers), rather than by the functionality of the recording system. However, the feasibility of transferring large datasets over the internet will depend on file size and upload speed. To reduce the risk of data loss and long upload times, data were uploaded after every block (i.e., 6–9 min of continuous recording at 90 Hz, resulting in files of approximately 9–17 MB). However, the resources provided within the onlineVR-toolbox are flexible with respect to upload frequency. We recommend evaluating file sizes and upload times during piloting to determine an appropriate batch size and uploading strategy.

**Analysis and results of VR task.** All data were pre-processed and analysed in the R statistical programming language (version 4.2.2; R Core Team, 2022) using RStudio (version 2023.06.2; RStudio Team, 2023). Visualisations were performed using the ggplot2 package (Wickham, 2016), and standard errors in all plots were calculated within subjects (Morey, 2008). Data and code for all analysis can be found at OSF (osf.io/27yjr).

Spontaneous memory usage was quantified through a metric we refer to as “attributes used in WM” (Draschkow et al., 2021; Kumle et al., 2024, 2025), which was calculated by counting the correct pick-ups and placements of target objects in between encoding from the model (see *Supplementary Notes 2* for a detailed description on this metric and associated analysis). Essentially, this metric indexes how many task-relevant steps were executed consecutively based on information in memory.

We successfully replicated previous lab-based findings on the estimates of spontaneous memory usage and the effect of movement effort (Draschkow et al., 2021; Kumle et al., 2024) within our online sample. Specifically, participants used more attributes in WM when movement effort was high (90°) compared to low (45°) (*t* =  − 5.48, *df* = 29, *p* < 0.001; Fig. 2D and E). When movement effort was low (45°), participants most often used only one attribute in memory (49.4%). In contrast, when movement effort increased (90°), participants relied on two attributes in memory (48.3%) in most cases (also see Table S1).

**Participant feedback.** Our primary interest in participant feedback was their experience navigating from Qualtrics to the task on Steam and back, since this step introduced a potential friction point: participants might struggle to find the task on Steam or misunderstand the instructions. However, feedback regarding the Steam game store was overwhelmingly positive. Most (87.5%) participants agreed or strongly agreed that “navigating to Steam was easy”, while only 5% disagreed or strongly disagreed. This aligns with direct messages received during testing: although some participants contacted us because they encountered technical difficulties (as noted in recruitment description), none were related to navigating to Steam.

One challenge specific to our task which was highlighted by multiple participants was positioning the VR environment within their physical space. However, participants reported being able to resolve these issues independently, often by restarting the task or adjusting their setup. That is, participants were able to draw upon their expertise regarding their own technical setup and were not discouraged by minor technical difficulties.

## Discussion

Virtual reality offers an unparalleled combination of experimental control and external validity, but its use has traditionally been limited to lab-based settings, constraining sample diversity and scalability. Remote VR data collection offers a promising solution for studying behaviours in large samples and diverse settings, but implementing online VR studies presents challenging technical hurdles. To address this challenge, we provide a comprehensive introduction to online VR experimentation, alongside a step-by-step tutorial for converting a lab-based VR task into a remotely deployable study using Unity and Steam. Additionally, we share an open-source onlineVR-toolbox containing code, documentation, and practical Notebooks designed to overcome the most pressing implementation challenges, particularly those related to data handling and transfer. Finally, we demonstrate the feasibility of this approach through a proof-of-concept case study replicating a well-established cognitive effect in a remote sample.

In this tutorial, we have deliberately focused on an approach that builds upon established frameworks, enabling researchers to build onto their existing expertise while maximising flexibility in the implementation of VR tasks. However, as outlined, this approach currently introduces certain friction points, as not all incorporated solutions are designed with research in mind (e.g., distribution through Steam). These include an initial 30-day waiting period after registering with Steam, and the manual implementation of solutions for counterbalancing and matching of participant IDs. We anticipate approaches to online VR experimentation to develop further going forward, for example, as new research-focused distribution services or additional dedicated end-to-end solutions become available. These developments may, in turn, inform and shift best practices in the field. In the meantime, we hope our overview helps researchers make an informed decision about which approach to remote online VR best suits their use case.

Importantly, we only covered solutions that allow for fully immersive studies (i.e., allowing participants to move through a virtual environment). However, using the VR headset as a “simple” screen may offer a promising compromise between implementation difficulty and experimental control for online data collection (see Loetscher et al., 2023, for a case study). That is, using a VR headset as a screen still allows us to control the participant’s field of view and to collect rich behavioural data, for example, from hand movements; however, it can be implemented using tools established for screen-based online studies (e.g., PsychoPy and WebGL). Further, our resources are targeted for unsupervised setups where participants are tested individually (as opposed to multiple participants interacting within a shared virtual environment; see Caruana et al., 2021; Rubo, 2025; Son & Rubo, 2025), but solutions for remote multi-user or supervised VR data collection are starting to become available (e.g., Steed et al., 2022).

Lastly, despite the overall promise of online VR data collection, certain limitations remain. Similarly to screen-based online studies, VR online studies are currently limited to behavioural measures since most consumer-grade VR headsets do not include eye-tracking capabilities. Nevertheless, the immersive nature of VR studies allows us to record exceptionally rich behavioural measures that go beyond what is possible with screen-based studies (e.g., including head and hand movements). Further, researchers should consider potential limitations stemming from hardware diversity. In this tutorial and case study, we developed the application to be compatible with all Steam-supported headsets, resulting in participants completing the study using a range of consumer-grade VR devices. This strategy is advantageous for maximising the eligible pool of participants. However, differences between headsets (e.g., field-of-view, resolution, or controller specifications; see also Angelov et al., 2020; Lynn et al., 2020; Mehrfard et al., 2019; Sauer et al., 2022) may be critical for studies that rely on precise visual stimulation, perceptual measurements, or device-specific manual responses. In such cases, researchers may consider standardising hardware by restricting participation to a specific headset model or statistically modelling this source of variance (e.g., including headset type as a random effect).

Finally, while remote VR studies make it easier to collect large samples, there is ongoing debate about how representative these samples truly are. Participation is limited to individuals who own a VR headset, which may constitute a specific subpopulation with distinct interests and characteristics (for a discussion of demographics of VR headset owners, see Kelly et al., 2021; Radiah et al., 2021). While concerns about representativeness are not unique to remote VR studies (e.g., Hanel & Vione, 2016; Henrich et al., 2010), researchers should consider how VR-specific sampling limitations may influence generalisability, particularly for research areas where demographic or experiential factors are likely to play a role. In general, it will be important to continue monitoring these limitations, as they may evolve rapidly with advances in headset technology (such as the integration of eye-trackers) or increased affordability.

In summary, our approach highlights not only the technical feasibility of online VR but also the exciting new opportunities for scalable, high-quality behavioural research. We hope our resources empower researchers to take their VR experiments beyond the lab.

## Supplementary Information

Below is the link to the electronic supplementary material.

Supplementary file1 (PDF 186 KB)

Supplementary file2 (MP4 16587 KB)

Supplementary file3 (MP4 27577 KB)

## Data Availability

Practical notebooks can be found at https://lkumle.github.io/onlineVRtoolbox_tutorials/. Materials for the onlineVR-toolbox are available at https://github.com/lkumle/onlineVR-toolbox. Data and materials for the case study can be found at osf.io/27yjr.

## Code availability

Code for the onlineVR-toolbox is available at https://github.com/lkumle/onlineVR-toolbox.

Analysis code for the case study is available at osf.io/27yjr.
