# Supplementary material for: Running virtual reality experiments online: A brief introduction and tutorial
Source: Behav Res Methods. 2026 Apr 29;58(6):148. doi: 10.3758/s13428-026-03025-w (PMC13128755; doi:10.3758/s13428-026-03025-w)
Supplement: Supplementary file 1 — Supplementary file1 (PDF 186 KB) [file 13428_2026_3025_MOESM1_ESM.pdf]

Supplementary Notes 1

Virtual environment

The virtual environment for the Object Copying Task consisted of a 300 x 300cm room containing the three task relevant stations: model, resource pool, and workspace. The model display consisted of 18 white squares (arranged in a grid of 3 rows of 6 placeholders), eight of which displayed target objects. Target objects were randomly displayed in one of 4 rotations (0°, 90°, 180°, 270°). In the resource pool, participants would find 24 cubes (10 x 10 cm) arranged in three rows of 8 cubes, including the 8 targets seen in the model and an additional 16 distractors. The cubes as well as target slots in the model were overlaid with images of objects from the Novel Object and Unusual Name Database (Horst & Hout, 2016). The workspace consisted of an empty grid of 18 white placeholders reflecting the configuration of the model display. Above the model, a timer counting backwards from 45 seconds was displayed. Participants always started a trial facing the model.

Participants were able to grab, move, and place cubes by pulling, holding, and releasing the trigger button on their controller. Correctly placed objects “locked into” the workspace with green contours indicating correct placement. The rotation of objects was irrelevant for placement in the workspace. In case of an erroneous placement (i.e., attempting to place a non-target object or placing a target object at a wrong location), the placeholder would light up red and the object would not lock into the workspace, nudging participants to correct their mistake. Participants were able to look back at the model before correcting their mistake but could not pick up another object until the mistake was corrected: all other objects in the resource pool disappeared until the incorrectly placed object was placed correctly or brought back to the resource pool.

Supplementary Notes 2

Analysis

Quantification of "attributes used in WM" (Draschkow et al., 2021; Kumle et al., 2024, 2025) was based on segmenting task behaviour into sequences, each of which started and ended with sampling information from the model (see **Figure 2B and C).** For example, a sequence could consist of sampling from the model, followed by picking up an object from the resource pool before concluding with looking back to the model, which would mark the end of the current and beginning of the next sequence. We therefore first identified periods during which participants looked at the model based by intersecting the head vector (i.e., unit vector originating from the headset's position and extends forward in the direction in which the participants head is facing) with the model. Since the successful copying of one object minimally requires the usage of two representational attributes (the object's identity for finding and picking it up, and its location for correctly placing it), *attributes used in WM* was then calculated by counting the correct pick-ups and placements of target objects within each sequence.

We then excluded the first sequence in each trial, as participants started each trial facing the model and had to first orient themselves within the task environment. We further excluded sequences if the detected model viewing period at the start of the sequences was < 50 ms or more than 3.5 standard deviations (SD) above a participant's overall mean model viewing period (1.4% of sequences excluded). For each participant, we then calculated means of attributes used in WM within the two movement-effort conditions. To test whether higher movement effort led to greater use of WM attributes, we conducted a one-sided paired-sample t-test comparing subject means between the two movement effort conditions. Additionally, we computed the probability of different numbers of attributes used in memory. Differences in means of the probability of different numbers of attributes used in WM between movement effort conditions were analysed using planned paired pairwise t-tests (see Table S1). We report Cohen's d as an effect size measure using the effectsize package (Ben-Shachar et al., 2020).

Table S1. Pairwise comparisons between the probability of using different number of attributes in memory between movement effort conditions (45° vs 90°).

Comparison	t	df	p	Mean diff	Cohen's d with 95% CI
1 attribute: 45° vs 90°	7.69	29	< 0.001	14.52	1.4 [0.89,1.91]
2 attributes: 45° vs 90°	-6.59	29	< 0.001	-12.88	-1.2 [-1.67,-0.72]
3 attributes: 45° vs 90°	-0.40	29	0.69	-0.36	-0.7 [-0.43, 0.29]
4 attributes: 45° vs 90°	-2.99	29	0.006	-2.35	-0.55 [-0.93, -0.16]
